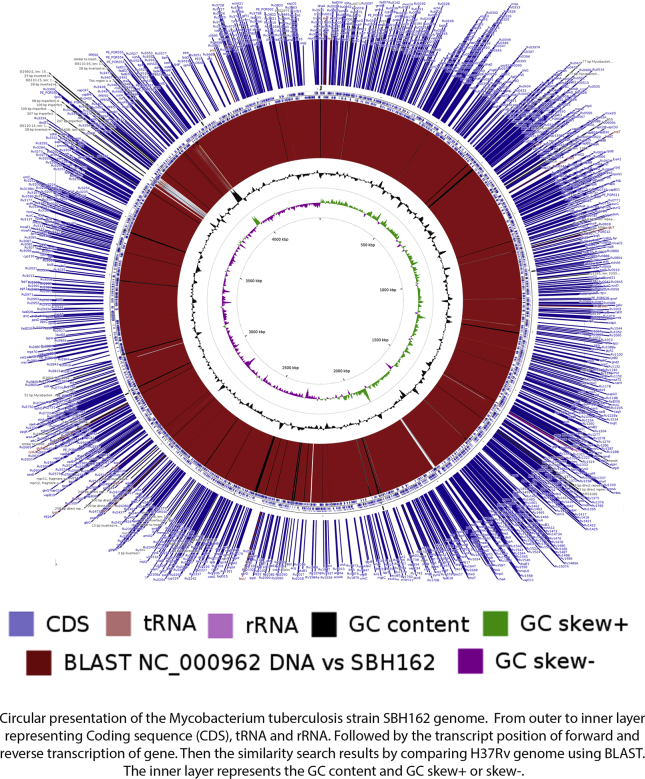# Whole genome sequencing data and analysis of a rifampicin-resistant *Mycobacterium tuberculosis* strain SBH162 from Sabah, Malaysia

**DOI:** 10.1016/j.dib.2019.104445

**Published:** 2019-08-28

**Authors:** Jaeyres Jani, Zainal Arifin Mustapha, Norfazirah Binti Jamal, Cheronie Shely Stanis, Chin Kai Ling, Richard Avoi, Naing Oo Tha, Valentine Gantul, Daisuke Mori, Kamruddin Ahmed

**Affiliations:** a Borneo Medical and Health Research Centre, Faculty of Medicine and Health Sciences, Universiti Malaysia Sabah, Sabah, Malaysia; b Department of Medical Education, Faculty of Medicine and Health Sciences, Universiti Malaysia Sabah, Sabah, Malaysia; c Department of Biomedical Sciences and Therapeutic, Faculty of Medicine and Health Sciences, Universiti Malaysia Sabah, Sabah, Malaysia; d Department of Pathobiology and Medical Diagnostics, Faculty of Medicine and Health Sciences, Universiti Malaysia Sabah, Sabah, Malaysia; e Department of Community and Family Medicine, Faculty of Medicine and Health Sciences, Universiti Malaysia Sabah, Sabah, Malaysia; f Tuberculosis and Leprosy Control Unit, Sabah State Health Department, Kota Kinabalu, Sabah, Malaysia

**Keywords:** *M. tuberculosis*, Whole genome sequencing, Next generation sequencing, Rifampicin resistant, Sabah, Malaysia

## Abstract

A *Mycobacterium tuberculosis* strain SBH162 was isolated from a 49-year-old male with pulmonary tuberculosis. GeneXpert MDR/RIF identified the strain as rifampicin-resistant *M. tuberculosis*. The whole genome sequencing was performed using Illumina HiSeq 4000 system to further investigate and verify the mutation sites of the strain through genetic analyses namely variant calling using bioinformatics tools. The *de novo* assembly of genome generated 100 contigs with N50 of 156,381bp. The whole genome size was 4,343,911 bp with G + C content of 65.58% and consisted of 4,306 predicted genes. The mutation site, S450L, for rifampicin resistance was detected in the *rpoB* gene. Based on the phylogenetic analysis using the Maximum Likelihood method, the strain was identified as belonging to the Europe America Africa lineage (Lineage 4). The genome dataset has been deposited at DDBJ/ENA/GenBank under the accession number SMOE00000000.

**Table:** Specifications Table

Subject area	Environmental Science
Specific subject area	Immunology and Microbiology
Type of data	Whole genome sequence with gene annotation and comparative genomic of *Mycobacterium tuberculosis* strain SBH162. The strain is also resistant to rifampicin drug.
Data acquisition	*De novo* whole genome sequencing, phylogenetic and variant calling with Illumina HiSeq 4000 system
Data format	Raw and analyzed data of whole genome sequences
Experimental factors	Isolated and cultured in 7H9 middlebrook medium, and incubated at BACTEC MGIT 320, Extraction of genomic DNA from a pure culture, library preparation for sequencing, Illumina sequencing, *de novo* assembly, annotation, variant calling and comparative genomic analyses
Experimental features	DNA extraction was performed using Masterpure Complete DNA and RNA purification kit; library was prepared using NEBNext® Ultra™ DNA Library Prep Kit for Illumina®; sequencing was performed using Illumina Hiseq 4000 system. The genome was assembled using SPAdes, variant calling by GATK tools, annotated with NCBI Prokaryotic Genome Annotation Pipeline and comparative genomic through kSNP3.
Data source location	Kota Kinabalu, Sabah, Malaysia
Data accessibility	Data is publicly available at NCBI Genbank from the following links: http://www.ncbi.nlm.nih.gov/bioproject/PRJNA524470 https://www.ncbi.nlm.nih.gov/biosample/SAMN11026786 https://www.ncbi.nlm.nih.gov/nuccore/SMOE00000000

**Table:** 

**Value of the data** • The data will shed light on the molecular biology of a *Mycobacterium tuberculosis* strain, which will be beneficial to researchers working on tuberculosis. • The data will give insight into drug resistance in *M. tuberculosis*, which will benefit clinicians and patients. • The data will help to understand the relation between *M. tuberculosis* strains from Sabah and other areas, which will contribute to policy making for the control of tuberculosis.

## 1 Data

**Fig. 1:**
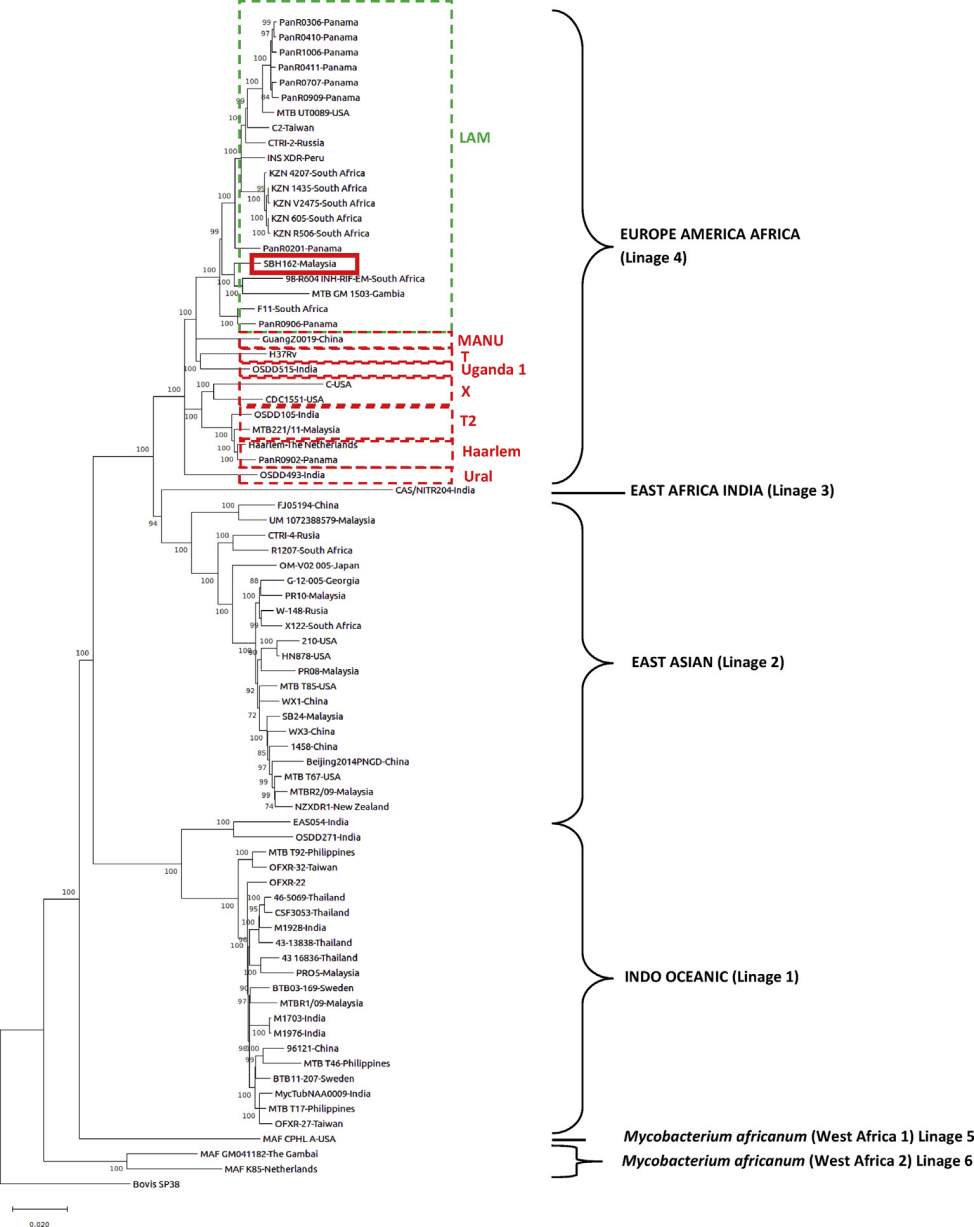
Comparative phylogenetic analysis of strain SBH162. This strain belongs to Lineage 4 and is clustered with other strains from the LAM family. The Malaysian strains are also in Lineage 4 and belong to T2 family while other Malaysian strains belong to Lineages 1 and 2. The phylogenetic tree was constructed using SNPs data extracted from the genome sequence. The phylogenetic tree was inferred using the Maximum Likelihood method and General Time Reversible model. The tree is rooted with *M. bovis* SP38 as outgroup.

In this paper, we present the data and analysis of the whole genome sequence (WGS) of *M. tuberculosis* strain SBH162 from Sabah, Malaysia. Tuberculosis was newly detected in a 49-year-old male patient using GeneXpert MDR/RIF. The whole genome was sequenced and *de novo* assembly, variant calling and comparative genomic of strain were performed. The *de novo* assembly of genome generated 100 contigs with N50 of 156,381bp. The whole genome size was 4,343,911 bp with G + C content of 65.58% and consisted of 4,306 predicted genes. In addition, the variant calling verified the mutation site in the *rpoB* gene, locus S450L. Based on the comparative genomics analysis using WGS of 77 strains, we determined that our strain belongs to the LAM family of Lineage 4 and is similar to the strains from South Africa [9] and Gambia [10] (see Fig. 1).

## 2 Experimental design, materials and methods

### 2.1 Isolation, culture, DNA extraction, library preparation and sequencing

The *M. tuberculosis* strain SBH162 was isolated from the sputum of a 49-year-old male from Kota Kinabalu, Sabah, Malaysia, who was newly diagnosed with tuberculosis in April 2017. The sputum was analyzed using GeneXpert MDR/RIF and cultured in 7H9 middlebrook medium using BACTEC MGIT 320 (Becton-Dickinson, Oxford, United Kingdom). Genomic DNA was extracted using Masterpure Complete DNA and RNA purification kit (Epicenter, Inc., Madison, Wisconsin, USA) according to the manufacturer's instructions. The quality of the extracted DNA was determined by Nanodrop 2000c spectrophotometer (ThermoFisher Scientific, USA). In addition, the concentration was determined using Qubit® 2.0 fluorometer (Invitrogen, ThermoFisher Scientific, USA).

### 2.2 Quality trim, *de novo* assembly and annotation

The genome was sequenced until 99% completion using 332X sequencing coverage. A total of 9,773,850 paired reads (∼1GB) of a 300-bp insert-size library by NEBnext Ultra kit (Illumina, San Diego, CA) were generated from Illumina HiSeq 4000. The data sequence was deposited in the Sequence Read Archive (SRA) (biosample accession number SAMN11026786) under the bioproject accession number PRJNA524470. For the purpose of analysis, the quality of the sequence read was checked using FastQC. All of the raw reads were pre-processed using BBMap version 38.43 tools [1], whereby the adapters were trimmed and the reads with less than 50bp were removed, based on the phred with a quality below Q30 using BBDuk.sh [1]. *De novo* assembly was performed using SPAdes version 3.11.1 [2]. The generated contigs were annotated using NCBI Prokaryotic Genome Annotation Pipeline (PGAP) [3].

### 2.3 Assembly statistic

**Table:** 

Sequencing depth	332X
Total length of sequences (bp)	4,343,911
Total number of contigs	100
N50 (bp)	156,381
GC (%)	65.58
CDSs	4,306
tRNAs	45
5s,16s,23s rRNA	1, 1, 1

Supplementary data 1, 2 and 3.

.

## 3 Variant calling

In the variant calling, sequence reads were trimmed with a phred score above Q20. Reads shorter than 50bp and possible contaminating adaptor sequences were excluded using BBMap version 38.43 tools [1]. Paired-end raw reads were mapped to the *M. tuberculosis* H37Rv reference genome (GenBank accession number NC_000962.3) using BWA MEM version 0.7.1231 [4]. Samtools version 0.1.1932 [5] was used to convert the SAM-BAM format and to sort the mapped sequences. Local realignment of the mapped reads was performed using GATK version 3.4.033 [6]. The statistic reports for the variant calling were generated using GATK and Samtools, whereby the average mapping rate of the sequences was 99.47% to the reference genome. Variant sites were filtered based on the following criteria: mapping quality greater than 50bp; base quality or base alignment quality greater than 20bp; and more than 10 covering each site. The SnpEff version 4.134 [7] was used for single nucleotide polymorphism (SNP) annotation. The list of SNPs (novel and previously reported) is provided as supplementary data 4.

This study was approved by the ethics committee at the Faculty of Medicine and Health Sciences, Universiti Malaysia Sabah (JKEtika 2/16 (6)).

## 4 SNP-based phylogenetic genotype study of SBH162

The genotype of our isolate was determined by the whole genome SNP. We identified that SBH162 belongs to Lineage 4 (LAM family) of the *M. tuberculosis* complex, where the sample was clustered with *M. tuberculosis* 98-R604 INH-RIF-EM and GM 1053 [8], [10], [14]. A mutation, S450L, was detected in the *rpoB* gene of our strain, which is responsible for resistant to rifampicin [13], [15]. Strain 98-R604 is from South Africa [9] and is resistant to isoniazid, rifampicin and ethambutol. On the other hand strain MTB GM1503 is from Gambia [14], is not rifampicin resistant *M. tuberculosis*.

Core-SNP was identified using kSNP3 package [11]. The entire SNP matrix was used in the phylogenetic analysis, which was performed with the Maximum Likelihood method using MEGA (Molecular Evolutionary Genetic Analysis) Software 6.0 [12] after aligning the nucleotide sequences using CLUSTAL W [12]. The significance of the branching patterns was evaluated through bootstrap analysis of 1,000 replicates. The whole genome sequence of 77 strains of *M. tuberculosis* were extracted from GenBank and used in the phylogenetic analysis [9], [10], [14].

## 5 Nucleotide sequence accession number

The whole genome sequence has been deposited at DDBJ/ENA/GenBank under the accession number SMOE00000000.

## Figures and Tables

**figs1:**